# Supplementary material for: Synergistic Effect of Nilavembu Choornam–Gold Nanoparticles on Antibiotic-Resistant Bacterial Susceptibility and Contact Lens Contamination-Associated Infectious Pathogenicity
Source: Int J Mol Sci. 2024 Feb 9;25(4):2115. doi: 10.3390/ijms25042115 (PMC10889799; doi:10.3390/ijms25042115)
Supplement: Supplementary file 1 [file ijms-25-02115-s001.zip › ijms-2774492-supplementary.pptx]

## Slide 1
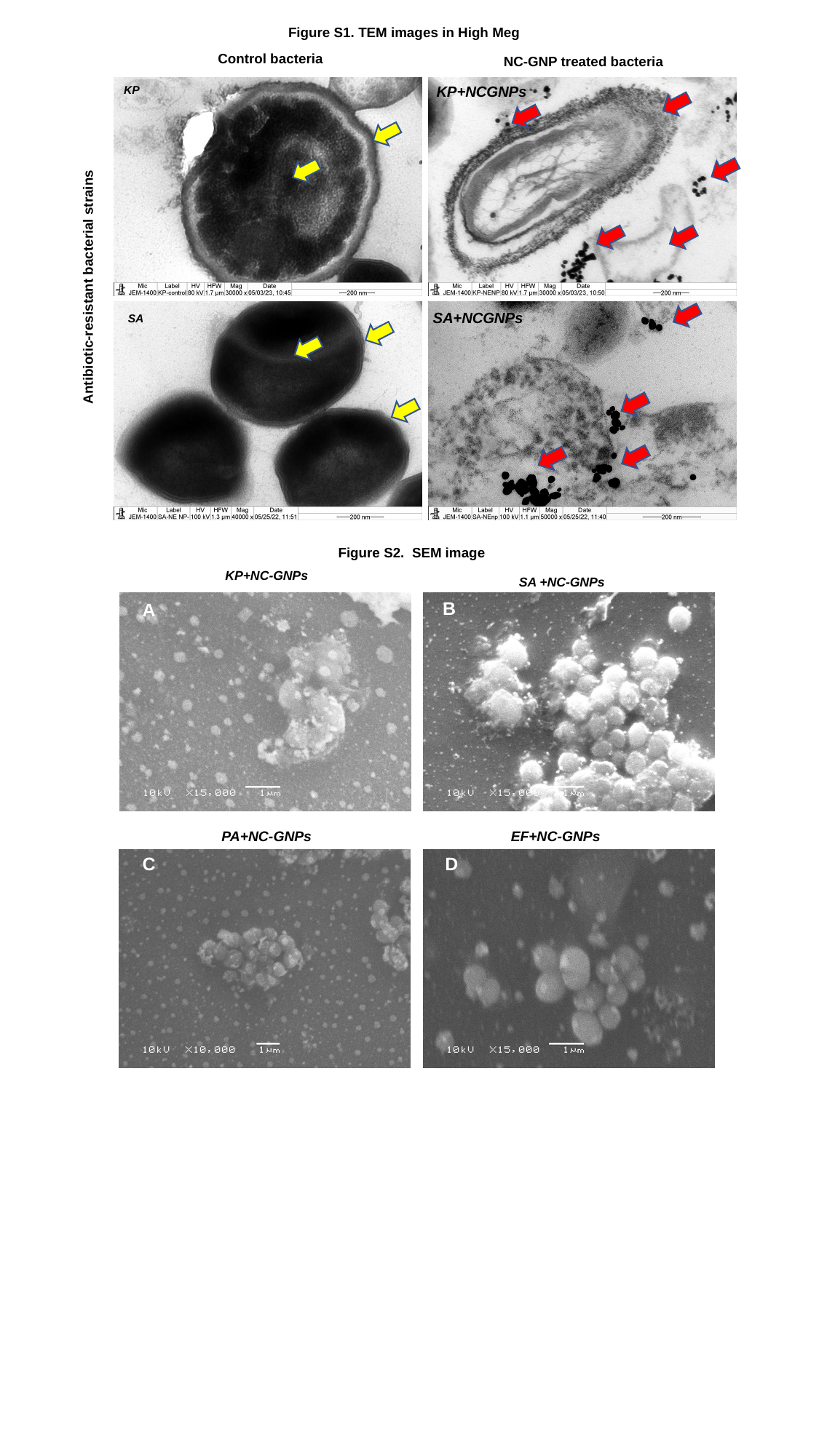

Figure S1. TEM images in High Meg
Control bacteria
NC-GNP treated bacteria
KP
KP+NCGNPs
Antibiotic-resistant bacterial strains
SA+NCGNPs
SA
Figure S2. SEM image
KP+NC-GNPs
SA +NC-GNPs
B
A
EF+NC-GNPs
PA+NC-GNPs
C
D
